# Not Another Caffeine Effect on Sports Performance Study—Nothing New or More to Do?

**DOI:** 10.3390/nu14214696

**Published:** 2022-11-07

**Authors:** Jason Tallis, Lucas Guimaraes-Ferreira, Neil D. Clarke

**Affiliations:** Research Centre for Sport, Exercise and Life Sciences, Coventry University, Coventry CV1 5FB, UK

**Keywords:** caffeine, sports performance, future research directions

## Abstract

The performance-enhancing potential of acute caffeine consumption is firmly established with benefits for many aspects of physical performance and cognitive function summarised in a number of meta-analyses. Despite this, there remains near exponential growth in research articles examining the ergogenic effects of caffeine. Many such studies are confirmatory of well-established ideas, and with a wealth of convincing evidence available, the value of further investigation may be questioned. However, several important knowledge gaps remain. As such, the purpose of this review is to summarise key knowledge gaps regarding the current understanding of the performance-enhancing effect of caffeine and justify their value for future investigation. The review will provide a particular focus on ten research priorities that will aid in the translation of caffeine’s ergogenic potential to real-world sporting scenarios. The discussion presented here is therefore essential in guiding the design of future work that will aid in progressing the current understanding of the effects of caffeine as a performance enhancer.

## 1. Introduction

The performance-enhancing effect of acute caffeine ingestion has been well established, with a number of meta-analysis demonstrating benefits for muscular strength and power [1,2,3,4], endurance performance [5,6,7,8], anaerobic power [9], sport-specific skills [10,11] and cognitive function [12]. Evidence within this field is now so vast, that a meta-evaluation of meta-analyses has been conducted, further supporting caffeine’s ergogenic effects [13]. With a wealth of convincing evidence available to support the benefits of caffeine consumption for exercise performance, the value and potential impact of further work in this area may be questioned. Despite this, the annual number of peer-reviewed publications in this field continues to grow, with a title search of “Caffeine” and “Sport” or “Performance” in PubMed showing near exponential growth in published articles. Despite the wealth of evidence, important knowledge gaps remain, and the purpose of this review is to outline important areas in need of future exploration and justify their value for investigation. Such work is essential in guiding the design of future work that will aid in progressing the understanding of the effects of caffeine as a performance enhancer, where the translation of findings to real-world scenarios should be prioritised. Focusing primarily on work that has evaluated the effect of caffeine on human performance, we have summarised the need for future work into ten priority areas, discussed herein.

## 2. Dose–Response Effect of Caffeine

It is generally considered that there is no dose–response relationship between the quantity of caffeine consumed and the magnitude of ergogenic benefit. Such conclusions are likely drawn based on the results of meta-regression, where caffeine dose has been shown to not influence the level of performance-enhancing benefit [6,7]. Whilst it is of value to explore moderators influencing the effect of acute caffeine consumption, conclusions drawn from such approaches may be misleading, given the number of interacting factors that have been proposed to influence caffeine’s effect on sports performance. For example, method of administration, circadian variation, mode of exercise, participant training status, habitual caffeine consumption and genetic variation have all been suggested to influence caffeine’s effect [7,14,15,16,17], and are regularly cited to rationalise conflicting results. The dose–response relationship between acute caffeine ingestion and performance benefit can only be accurately determined via studies that have directly examined the effects of different caffeine doses on matched experimental procedures. Surprisingly, studies in this area are sparse.

Of the small number of studies to directly address this issue, a number fail to show a benefit of caffeine at any of the experimental doses investigated [18,19,20], and whilst these are important in developing the general understanding of caffeine as a performance enhancer, they reveal little regarding the dose–response relationship. Of the remaining studies, a number show effects at a single dose which is not replicated in others [21,22,23,24], and in some specific cases, a similar level of benefit across doses [25,26,27,28,29,30]. Direct comparison between previous works is limited by differences in the doses examined, the population assessed and differences in exercise modality. Thus, the current understanding of the dose–response effect of acute caffeine ingestion is poor. 

**Figure 1:**
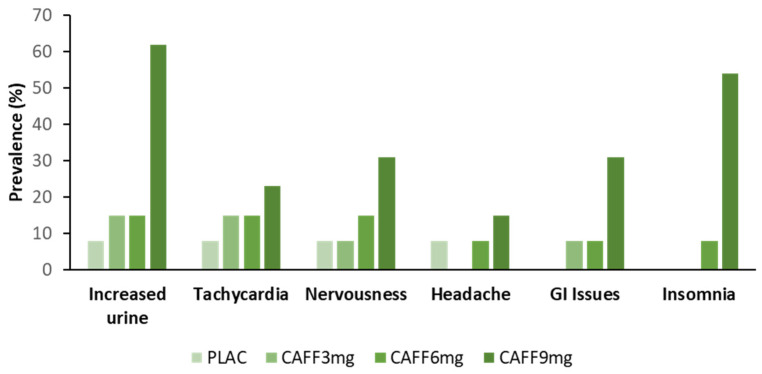
Percentage of participants reporting side effects to increasing caffeine dose following a neuromuscular test battery. Adapted from Pallarés et al. [25]. [PLAC = placebo; CAFF = caffeine; GI = Gastrointestinal; results based on data from N = 13 resistance-trained men].

Moreover, evidence suggests that the relationship between dose and response is more complex than the magnitude of benefit elicited. For example, Tallis and Yavuz [26] demonstrated that the ingestion of both 3 mg.kg^−1^ and 6 mg.kg^−1^ caffeine caused an equivalent increase in the maximal voluntary isokinetic force of the concentric knee extensors, however, only 6 mg.kg^−1^ improved performance over sustained contractions. These findings are further supported by work from Pallarés et al. [25], who indicated that the acute ingestion of 3, 6 and 9 mg.kg^−1^ of caffeine caused an equivalent increase in squat and bench press bar velocity and propulsive power at low loads, however, at heavier loads, only the higher doses elicited a performance-enhancing benefit. In fact, bench press bar velocity and squat peak power at the highest load (90% 1 repetition maximum), as well as maximal cycle peak power output were only increased with the 9 mg.kg^−1^ dose, despite a higher prevalence of side effects (Figure 1). Such evidence suggests that a dose–response effect of caffeine is likely, but is more binary in nature where an effect is either prevalent or not.

It is evident that the understanding of caffeine’s dose–response effect warrants further investigation. Where the relationship between caffeine dose and performance is binary, future work is needed to understand caffeine dose requirements to elicit benefits in different facets of physical performance and exercise modalities. Moreover, the lack of direct evidence should prevent a potential relationship between caffeine dose and magnitude of performance-enhancing benefit from being discounted. The potential for a dose–response relationship is evident in a recent meta-analysis, demonstrating that acute caffeine ingestion elicits an improved rate of force development during resistance exercise, with the magnitude of the measured benefit increasing at a higher dose [4]. Albeit very specific and limited in quantity, there is some direct support for a dose–response effect in the literature [23,31,32]. However, recent work by Filip-Stachnik et al. [33] is the most convincing evidence to date, with data indicating superior improvements in bench press 1-RM in resistance-trained females following 6 mg.kg^−1^ caffeine consumption compared to 3 mg.kg^−1^.

The debate regarding the optimal dose of caffeine to elicit performance-enhancing effects has widened recently with studies exploring the efficacy of low-dose caffeine. It has been well accepted for a number of years that 3 mg.kg^−1^ of caffeine ingestion is the minimum dose required to elicit improved physical performance [34]. However, there is a growing suggestion that doses lower than 3 mg.kg^−1^, and even as little as 1 mg.kg^−1^ [35], may be effective in enhancing some aspects of physical performance [36,37]. A recent meta-analysis indicates that caffeine doses between 1–2 mg.kg^−1^ may elicit similar benefits on vertical jump performance to that seen with higher doses [37]. In the same manner previously outlined, low-dose caffeine may be important in eliciting a binary caffeine response. For example, when effects of 2, 4 and 6 mg.kg^−1^ caffeine on muscular strength were examined in resistance-trained males, only 2 mg.kg^−1^ enhanced lower body strength and 4 and 6 mg.kg^−1^ was needed to elicit a small increase in upper body strength [32]. Conversely, 3 mg.kg^−1^ caffeine has been shown to increase maximal power in the half squat and bench press, effects that were not evident following 1 mg.kg^−1^ ingestion [38]. Whilst doses lower than 3 mg.kg^−1^ may elicit some task-specific benefits, evidence suggests a broader range of benefits occurs at doses of 3 mg.kg^−1^ [35].

Limited studies in this area and the lack of consistency in results highlight the need for future work evaluating the effects of low-dose caffeine consumption on sports performance across exercise modalities. Such work is particularly valuable given that doses lower than 3 mg.kg^−1^ more closely reflect doses consumed in commercially available products. Positive effects at lower doses would be valuable in managing potential habituation to caffeine’s effects but are also associated with reduced prevalence of side effects and disrupted sleep [39]. Furthermore, whilst in some instances, improvement in a single facet of physical performance may be effective in improving sporting outcomes, future work may wish to consider the effect of caffeine dose on multiple facets of performance. Waer et al. [23] demonstrated that low dose caffeine (100 mg) improved simple reaction time but had no effect on the physical function of healthy middle-aged women. However, the reverse was true when a higher dose (400 mg) was consumed. In addition doses as high as 9 mg.kg^−1^ may be needed to improve specific aspects of muscular strength [25], where the side effects seen at such doses [39] are likely to be detrimental to cognitive function.

## 3. Time Course Effects of Acute Caffeine Effects

Acute caffeine effects on sports performance are prevalent following the mode-specific optimal ingestion period, where studies typically evaluate caffeine’s performance-enhancing potential ~1 h post-ingestion. Whilst following this ingestion period there is evidence supporting benefits for both short-duration strength and power type activities, and longer duration endurance activities, the time course of caffeine’s ergogenic effects are not well understood. Developing an understanding of the time course of caffeine’s performance-enhancing effects would be of particular importance for athletes involved in long-duration activity or sports where events are spread over the course of a day. Furthermore, repeated dosing appears to be common practice in some sports such as soccer [40], however, the need for and the value of repeated dosing is not well understood. Whilst repeated dosing may be effective in inducing sustained benefits over longer durations, caffeine has a half-life of 1.5–9.5 h [41], highlighting a need to balance potential positive benefits with well-established detrimental side effects and impact on sleep which are more prevalent following the consumption of higher caffeine quantities [39].

The time course of caffeine’s ergogenic effects may be dependent on habitual caffeine consumption. Bell and McLellan [42] demonstrated that in non-caffeine users, time to exhaustion when cycling at 80% VO_2max_ was improved when 5 mg.kg^−1^ caffeine was consumed 1, 3 and 6 h prior to exercise. However, in a group of regular caffeine users, performance was only improved when ingestion occurred 1 and 3 h prior to exercise. Whilst these results indicate that is may be possible to harness the beneficial effects of caffeine over an extended timeframe, they reveal little regarding the time course of the effects.

Negaresh et al. [43] compared the effect of a single high dose (10 mg.kg^−1^), moderate dose (4 mg.kg^−1^), a repeated dose (2 mg.kg^−1^ on 5 occasions), and an individualised repeated dosing strategy based on performance decrement on the Pittsburgh Wrestling Performance Test, assessed prior to five competitive matches. The first four matches were separated by 45 min, and 180 min separated the fourth and fifth matches. The single high-dose caffeine treatment improved performance prior to the first match, the repeated dose and individualised repeated dosing strategy improved performance prior to the third and fourth match, and only the individualised repeated dosing strategy improved performance prior to the fifth match. These findings indicate that whilst it may be possible to harness the performance-enhancing potential of caffeine a number of hours following ingestion, the effects of a bolus dose may be relatively short-lived. However, studies examining the efficacy of repeated dosing are lacking and controversial. For example, Conway et al. [44] concluded that a divided caffeine dose (3 mg.kg^−1^ provided 60 min prior to exercise and 45 min into exercise) provided no greater ergogenic benefit than a bolus dose (6 mg.kg^−1^) on cycling time trial performance. However, it should be noted that the benefits of a repeated dose were not prevalent until 150 min post the initial ingestion in the work by Negaresh et al. [43].

The time course of caffeine’s acute effect may also be influenced by the mode of administration. Caffeine gum has been shown to be effective for improving aerobic cycling performance 5 min post-ingestion, but with no effect when exercise commenced 60 min and 120 min post consumption [45]. These results are supported by a recent meta-analysis, suggesting that the benefits of caffeine gum consumption were prevalent when ingested within 15 min of exercise, but not at timeframes longer than this [46]. This is particularly intriguing given that a second spike in caffeine blood plasma concentration, caused by absorption through the gut, has been shown to occur 50 min post consumption of caffeinated gum [47].

Evidence in this area is generally lacking and further work is needed to understand the time course of acute caffeine effects on different modes of exercise, the efficacy of strategies for repeated dosing and how such effects may be moderated by habitual caffeine use. Future work may also consider the effect of sustained-release caffeine products. For example, Xtenergy™ has been shown to result in slower absorption but more sustained plasma caffeine concentrations compared to traditionally consumed caffeine in capsule form. Moreover, this slow-release caffeine product has been shown to prolong benefits on measures of mood and alertness [48]. Whether these effects extend to physical performance is yet to be investigated. One important point of note is that slow-release caffeine has been shown to reduce the peak plasma concentration [48] which may potentially reduce or prevent an ergogenic benefit. Future work may therefore consider experimenting with higher caffeine doses than those ingested via more traditional means. It may also be possible to harness similar effects to slow-release caffeine products by combining caffeine and carbohydrate ingestion [49]. Such approaches may be particularly beneficial for exercise modalities that rely on carbohydrate metabolism.

## 4. Effect of Alternative Modes of Caffeine Administration

**Figure 2:**
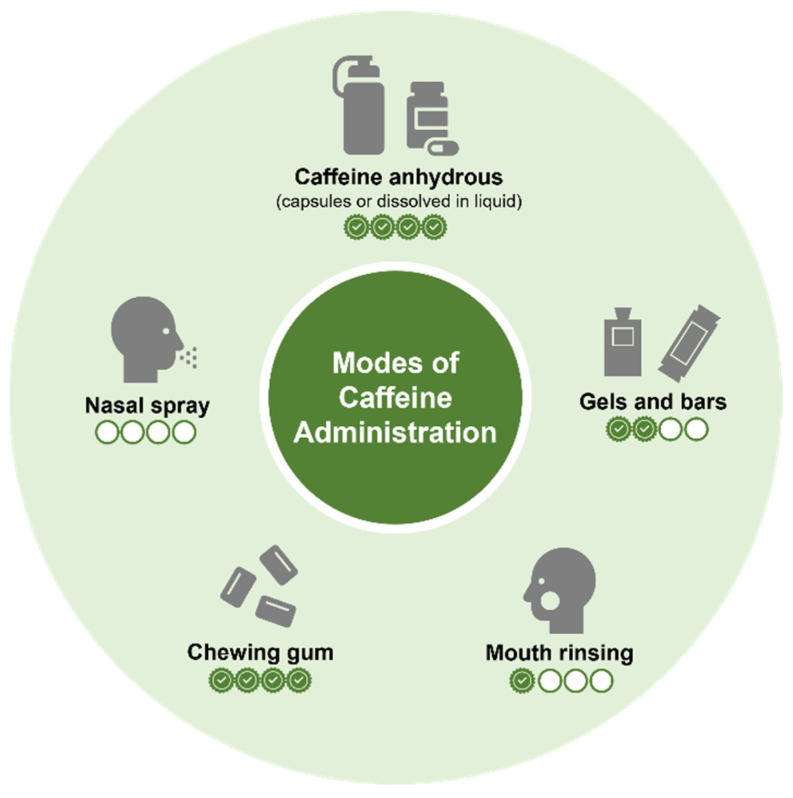
Summary of different modes of caffeine administration. [Symbols represent current level of supporting evidence based on expert opinion of the authors].

For the most part, the current understanding of caffeine’s acute effects on sports performance is derived from studies where participants consume caffeine anhydrous primarily in capsule form or that dissolved in liquid. However, more recent work has examined the ergogenicity of caffeine administered in alternative forms with studies investigating the effect of caffeinated chewing gum [46], nasal spray [50,51], mouth rinsing [52] and gels and bars [52,53,54]. While some of these forms warrant investigation given that they represent commercially available products, alternative methods of consumption may also provide distinct logistical and mechanistic advantages to traditional modes of caffeine consumption. Figure 2 illustrates the different modes of caffeine administration with their respective level of evidence based on investigations examining effects on physical performance.

Whilst there is clear inter-individual variation [55], and potential for genetic influence [56], peak caffeine blood plasma concentration typically occurs ~60 min post ingestion [57,58]. As such, studies that require participants to consume caffeine delivered in capsules or dissolved in fluid typically commence assessment of physical function this duration of time post ingestion [2,5,59,60]. In particular, the performance-enhancing potential of caffeinated chewing gum has received growing attention, given evidence supporting more rapid absorption and a faster onset of pharmacological effects. The release of caffeine from chewing gum as a result of maceration in the mouth has been suggested to be absorbed into the bloodstream via the highly vascular buccal mucosa in the oral cavity [61]. Furthermore, it has been suggested that caffeine mouth rinsing may elicit a performance-enhancing effect due to caffeine activating bitter taste receptors in the mouth [62], which may stimulate regions of the brain associated with information processing and reward [63]. It is firmly considered that the ergogenic effect of caffeine occurs due to its action as an adenosine receptor antagonist, promoting the increase in excitatory neurotransmitters [64,65,66]. Evidence demonstrates that adenosine receptors are prevalent in the oral cavity of mammals [67].

The consumption of caffeinated chewing gum has been shown to cause an initial spike in caffeine blood plasma concentration after ~10 min and a later second peak after ~50 min due to absorption in the gut [47]. A growing body of evidence has demonstrated positive effects of caffeine gum across a wide range of activities inclusive of endurance performance [45,68,69], anaerobic power [69,70], muscular strength and power [69,71,72] and cognitive function [73,74]. The performance-enhancing potential of caffeinated gum was confirmed in a recent meta-analysis [46], however, sub-analysis indicated that benefits were only prevalent when exercise commenced within 15 min of consumption, and effects were not prevalent in untrained participants.

The ergogenic potential of caffeine mouth rinsing, where caffeine is rinsed in the mouth for 5–20 s without ingestion, has also received recent attention. Mouth rinsing may hold particular benefits over other methods of administration, potentially mitigating issues with side effects of ingestion which may negatively influence performance. Attributed to similar mechanisms to the action of caffeinated chewing gum in the mouth, in a small number of cases caffeine mouth rinsing has been shown to elicit performance-enhancing effects on endurance activity [75,76], anaerobic exercise [77], muscular strength [78] and cognitive function [79,80]. However, the evidence is far less convincing when compared to the body of work examining the effects of caffeinated chewing gum with a recent systematic review demonstrating benefits in only 5 of the 15 included studies evaluating physical performance, with many of the included studies having low methodological quality [81]. It would appear based on the limited available evidence, that caffeine mouth rinsing may be more effective when repeated at regular intervals rather than a single rinse prior to the completion of the exercise protocol [52,81]. Given that caffeine mouth rinsing holds some performance-enhancing potential, further work in this area should be considered, focusing on the frequency, duration and dose–response effects of caffeine mouth rinsing. To date, only a single study has evaluated the dose–response effect of caffeine mouth rinsing, demonstrating that a repeated 5 s rinsing of a 3% (750 mg) caffeine solution increased the number of repetitions unit failure in the bench press and reduced RPE, with no effect demonstrated with a 1% (250 mg) or 2% (500 mg) dose [78]. Results summarised in the recent systematic review on this topic have only considered doses up to 2% [81]. As such, future work should consider the ergogenic effects of higher caffeine doses, and maybe at quantities higher than that typically used when caffeine is ingested, given that negative side effects associated with ingestion may not be prevalent.

There is both supporting [51,53,54,82,83] and conflicting evidence [50] regarding the ergogenic potential of caffeinated nasal sprays, dissolvable mouth strips, and caffeine bars and gels. The limited number of studies, lack of sport-specific context in a number of cases, and myriad of factors beyond the mode of administration influencing the performance-enhancing effects of caffeine prevent precise conclusions regarding effectiveness and best practice for use of these modes of administration. In particular, evidence regarding the effect of caffeinated nasal spray, dissolvable mouth strips and mouth aerosols is substantially lacking. With each mode showing potential, further work is warranted.

Despite a growing body of evidence supporting the use of alternative modes of caffeine consumption, studies directly comparing the effectiveness between modes are lacking. Irwin et al. [84] demonstrated comparable effects of low-dose (80 mg) caffeinated coffee, energy drink, capsule and dissolvable mouth strips on alertness, tiredness and choice reaction time. Whalley et al. [83] also demonstrated comparable effects of caffeinated chewing gum, dissolvable mouth strips and capsules (at doses of 3–4.5 mg.kg^−1^) in improving the time trial performance of amateur runners. While both studies offer important insight, their findings have important limitations. Importantly, a lack of treatment blinding may have had a substantial impact on the outcomes, particularly considering the well-reported performance-enhancing potential of caffeine expectancy [85]. Furthermore, irrespective of the mode of administration, the period between ingestion and assessment of the outcome variables was standardised at 15 min, which means the initiation of assessment would not have occurred at the point of peak plasma concentration for methods that work via absorption in the gut. Future work is therefore needed to more precisely directly compare the ergogenic effect of different modes of caffeine administration, considering a broader range of exercise intensities and modalities. Furthermore, no study to date has considered the potential of combining modes of administration, where it may be possible to harness specific benefits of each mode to elicit a greater effect. In some cases, such approaches may also better represent practices for caffeine consumption in sport, where evidence suggests prior to and during performance caffeine is consumed in a number of forms [40].

## 5. Influence of Caffeine Habituation and Genotype

The responses to caffeine are often variable and possibly affected by a number of factors such as genotype and habitual caffeine use [86]. There has been a long-standing paradigm that habitual caffeine intake may influence the ergogenicity of caffeine supplementation [87], however, in a recent meta-analysis Carvalho et al. [88] reported that habitual (i.e., chronic) caffeine consumption does not appear to influence the acute ergogenic effect of caffeine. Therefore, the ingestion of caffeine at doses of 3 to 6 mg·kg^−1^ body mass would appear to be beneficial for those with high and low habitual caffeine consumption.

Summary of the impact of habituation of caffeine’s performance-enhancing effects has been drawn from evidence from three different study designs. By measuring typical caffeine consumption habits prior to completion of the designed procedures, there is evidence to support the ergogenic potential of caffeine, or in some cases lack thereof [89], in participants that are habitual caffeine users [90,91,92,93]. Drawing summary from such work is somewhat limited by the number of moderating factors influencing the caffeine response. A more appropriate approach has been to use a between-subject design to measure the ergogenic response in participants stratified by typical caffeine consumption habits. Whilst these studies are limited in number, the general consensus is that caffeine is ergogenic irrespective of typical caffeine consumption habits [94,95,96,97,98]. For example, Clarke and Richardson [95] demonstrated that the level of habitual caffeine ingestion was not associated with the magnitude of improvement in 5 km performance following the ingestion of coffee providing 3 mg·kg^−1^ body mass of caffeine, a value below the habitual intake of the high-users. Similar findings have been reported by Grgic and Mikulic [98] in that the acute effects of caffeine supplementation on resistance exercise, jumping, and Wingate performance were not affected by habitual caffeine intake. Irrespective of these findings, results should still be interpreted with caution given that different thresholds of caffeine consumption are used to stratify groups, that groups are typically stratified by absolute caffeine consumption rather than that relative to body mass, given well-reported issues with accurately measuring caffeine consumption [99], and that several factors likely moderate caffeine’s effect which may influence between-group comparison.

Arguably, a within-subjects experimental design is more appropriate for evaluating the impact of habituation, where in this context, the ergogenic potential of caffeine is assessed prior to and following a period of prescribed caffeine ingestion. Studies that have adopted this approach are lacking, and findings more controversial with evidence to suggest that chronic supplementation fails to impact the magnitude of the acute caffeine response [100], whilst others show blunting effects [15,101]. The discrepancy in results can likely be attributed to differences in prescribed dose, duration (typically between 3–7 weeks), and habitual caffeine use prior to participation in the study. Future work should therefore continue to examine the impact of caffeine habituation on caffeine’s ergogenic potential with a specific focus on using precise measures of evaluating caffeine consumption habits of the recruited population, understanding if effects are dose specific, and examining the impact of caffeine prescribed over durations longer than the 3–7 weeks used in previous studies. Future work should also consider the impact of genotype on habituation, given the association between polymorphisms in ADORA2A, CYP1A2 and AHR genes and caffeine consumption habits [102,103,104]. Genotype has also been suggested to influence caffeine’s ergogenic effects.

Two of the genes suggested to have the largest impact on the ergogenicity of caffeine are CYP1A2 and ADORA2A [5]. Guest et al. [16] reported that caffeine improved endurance performance at a dose of 2 and 4 mg·kg^−1^ body mass for fast metabolizers of caffeine who have the CYP1A2 AA genotype. In contrast, among the slow metabolizers, there was either no effect (AC genotype) or impaired performance (CC genotype) under the same caffeine conditions. Several further studies support the impact of polymorphisms in the CYP1A2 gene on caffeine’s ergogenic effect, however, an equal number of studies indicate no influence [105]. Whilst polymorphisms in ADORA2A have been shown to influence caffeine sensitivity [106], studies evaluating the impact on exercise performance are sparse. Loy et al. [107] demonstrated that caffeine influenced cycling performance in carries of the TT allele but not in the CT/CC group. Conversely, the ADORA2A genotype did not influence caffeine’s effect on strength and power performance in a population of professional handball players [108]. Evidently, more work is needed to explore the influence of the ADORA2A genotype on sports performance along with other promising targets such as the HTR2A genotype which has been shown to influence caffeine’s ergogenic response to aerobic cycling [109].

There is some suggestion that genotype effects may be specific to exercise modalities and influenced by dose [105,108]. However, it is generally considered that work in this area is somewhat limited by small sample sizes, and a lack of breadth in exercise modalities explored [105]. Beyond this, future work should examine optimising ingestion periods based on CYP1A2 genotypes, were the standardised ingestion approach used in current studies may mask the potential ergogenic effects in slow metabolisers, who may need a longer ingestion period before performance-enhancing benefits occur. Other than in the work by Guest et al. [109], the focus of previous work in this area has been to evaluate the impact of a single genotype on caffeine’s ergogenic effects. Whilst this presents a considerable challenge, a substantial improvement in our understanding of the influence of genotype on caffeine’s ergogenic effect can only be made by stratifying groups by polymorphisms in multiple genes.

## 6. Caffeine as a Supplement to Augment Adaptations to Exercise Training

Given the well-established effects of acute caffeine ingestion on sports performance, it would seem intuitive that improved or maximised performance in a bout of exercise multiplied over the duration of an appropriately designed training regime may evoke a more pronounced adaptation. In light of limited direct evidence, such theory is likely the basis of the inclusion of high quantities of caffeine in pre-workout supplements. To date, only five studies have examined the potential of chronic caffeine consumption to augment adaptations to exercise training. Of these studies, only one has considered endurance exercise.

Using a between-subjects, double-blind, placebo-controlled study design, MALEK et al. [110] demonstrated that supplementation of 201 mg (~3 mg.kg^−1^) of caffeine 60 min prior to each training session, resulted in equivalent training-induced increases in VO_2peak_ and time to exhaustion (at 90% of VO_2peak_) following an 8-week endurance-focused training program (45 min treadmill running at 75% of the heart rate at VO_2peak_, three times per week) in a group of non-specifically trained male and female participant’s.

Conversely, Kemp et al. [111] demonstrated that in non-specifically trained participants, 3 mg.kg^−1^ of caffeine consumed prior to every session caused superior improvements in squat and bench press 1-RM compared to a placebo group fowling completion of a 6-week resistance training protocol (3 sessions per week; 4 sets repetitions until failure of the squat, deadlift, bench press and bench pull at 80% 1 RM). Using the same dose and a similar population, Giráldez-Costas et al. [112], demonstrated changes in bench press 1 RM following 4 weeks of resistance training (3 sessions per week: wks 1–2: 4 sets of 10 repetitions at 60% 1 RM; wks 3–4: 4 sets of 8 repetitions at 70% 1 RM) were similar between a placebo and caffeine group. However, participants in the caffeine group had more adaptations in lifting velocity post-intervention. Pakulak et al. [113] demonstrated no benefit of caffeine supplementation used prior to resistance training over a 6-week intervention. However, comparisons were made with only 5 participants in each of the caffeine and placebo groups.

In part, these previous studies are limited by low sample size, a failure to quantify if an acute caffeine effect is prevalent, and although a point of contention, a failure to consider the potential paradoxical impact of habituation to a repeated dose. Only a recent study by Tamilio et al. [100], examining the effect of pre-sessional ingestion of 3 mg.kg^−1^ caffeine in rugby union players over the course of a 7-week resistance training intervention (2 sessions per week; 2 sets repetitions until failure of squat, deadlifts, chest press, seated shoulder press, power clean, hang clean at 70% 1 RM, sit-ups and press-ups without any external load), has considered some of these factors. Despite caffeine eliciting beneficial effects on upper body and lower body isokinetic strength and an increase in total weight lifted over the course of the intervention, the improvement in muscular function post-intervention was equivalent between the caffeine and placebo groups. Habituation influencing the response was discounted given that acute caffeine effects on measures of muscular function post-intervention were still prevalent in the caffeine group. Whilst the training intervention used by Tamilio et al. [100] was effective in quantifying within and between session effects of caffeine and was effective in improving measures of muscular function post-training, fixed load repetitions until failure protocols are lacking in exercise progression, and as such, are likely not the most effective approach for training muscular strength and power. Furthermore, in most cases, such approaches are not representative of more complex strength and power training regimes adopted by athletes [114].

The small number of studies and ambiguous results examining the potential for caffeine to augment exercise training incite a need for further research in this area, considering different exercise modalities, a range of training methods and particular focus on measuring habituation to the prescribed dose. Such work should also consider the effect of caffeine as a supplement to enhance the effects of exercise training in a broader context considering caffeine’s effect on sleep [39] and the importance of sleep for recovery [115], that the ergogenic response may be specific to the time of day [116], and that a higher volume of work elicited as an acute effect of ingestion may exacerbate exercise-induced muscle damage [29].

## 7. Effect of Caffeine on Exercise-Induced Muscle Damage and Recovery

An important area of research that has received relatively little attention is the effect of caffeine on exercise-induced muscle damage (EIMD) and recovery. It has been proposed that caffeine ingestion could alleviate the delayed-onset muscle soreness (DOMS) [117,118], attenuate temporary loss of muscle function [119], and decrease blood markers of muscle damage [120] following damage-inducing exercise. However, as recently reviewed by our research group [121], the effects of caffeine ingestion on indirect markers of exercise-induced muscle damage are still limited and inconclusive. Reasons for conflicting results include methodological differences, such as methods to elicit muscle damage, muscle groups assessed, different experimental designs (i.e., between-subjects vs. within-subjects designs), caffeine supplementation protocol (i.e., single vs. repeated ingestion; dosage) and timing of caffeine intake in relation to the EIMD induction protocol. For example, it has been hypothesised that caffeine ingested prior to a muscle damage-inducing protocol could attenuate muscle damage; but when used after the induction of EIMD it could relieve the symptoms, including recovery of muscle function and pain perception, albeit acutely in the time where caffeine plasma concentration remains high following ingestion. Alternatively, caffeine ingestion pre-exercise could result in an increase in total work performed, potentially increasing EIMD. Not all studies that aim to assess the effects of caffeine on EIMD have an experimental design that allows identifying whether caffeine exerts a protective effect against EIMD, only relieve the symptoms during the recovery phase, or even results in greater muscle damage due to its ergogenic effect. Therefore, caution is needed when interpreting such studies and the methodological aspects must be carefully considered.

Indirect markers of EIMD include DOMS, blood markers (e.g., blood creatine kinase; lactate dehydrogenase) and muscle function, such as maximum voluntary isometric contraction (MVIC). Some studies have demonstrated that caffeine ingestion attenuated pain perception between 24 and 48 h following the muscle damage protocol [117,119,122,123]. However, other investigations found contrasting results, with no difference between caffeine and placebo [124], or higher DOMS in the caffeine group [29]. The effects of caffeine ingestion on blood markers of EIMD are also controversial. Ferreira et al. [120] found lower circulating CK levels in the caffeine group, while Stadheim et al. [29] and Bassini-Cameron et al. [125] found the opposite (higher CK levels after caffeine ingestion compared to placebo). However, most studies found no significant differences between placebo and caffeine groups on blood markers of EIMD (reviewed by Caldas et al. [121]). Finally, four studies investigated the effects of caffeine on MVIC following EIMD. Chen et al. [119] investigated the effects of caffeine (6 mg/kg) 24 and 48 h following an EIMD protocol consisted of 30 min of downhill running in 10 male and 10 female college athletes. Compared to placebo, caffeine ingestion resulted in a 10.2% higher MVIC 48 h after EIMD. However, most studies demonstrated no differences between caffeine and placebo conditions on MVIC recovery after EIMD [123,124,126].

**Figure 3:**
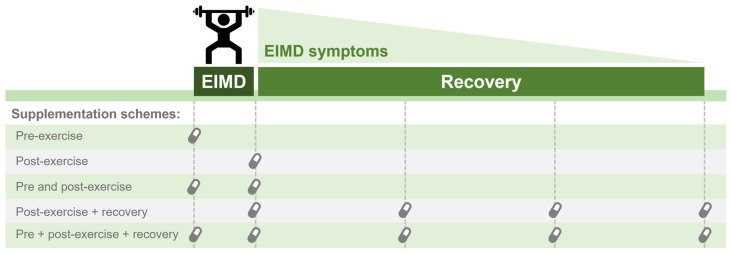
Representation of possible supplementation schemes to counteract exercise-induced muscle damage (EIMD) symptoms or accelerate recovery after EIMD.

Taken collectively, research suggests that caffeine ingestion may result in lower pain perception but its effects on strength recovery after EIMD is still controversial. As mentioned, differences in the EIMD protocol (e.g., matched for total work performed) and/or caffeine supplementation regime (e.g., timing; single or repeated doses) could explain the discrepancies among studies, and whether caffeine ingestion prior to exercise elicits a protective effect on EIMD, is still unclear. However, due to its acute effects on muscle performance and pain perception, caffeine consumption may be a valid strategy for recovery between strenuous exercise sessions. Future studies should further investigate the effects of caffeine ingestion at different times regarding EIMD (i.e., pre vs. post EIMD) and different supplementation protocols (e.g., dose, single vs. continuous supplementation, form of administration, etc.). Figure 3 illustrates different supplementation schemes that should be considered when interpreting studies on the effect of caffeine on EIMD. Additionally, a challenging but exciting area for study involves understanding the mechanisms of action that could be involved with the effect of caffeine on EIMD. Thus, it will be possible to understand whether caffeine can exert a protective effect (e.g., on a cellular level) against muscle damage, or simply act as an ergogenic aid, improving contractile function following EIMD.

## 8. Effect of Caffeine Expectancy on Exercise Performance

Further to the well-reported effect of acute caffeine consumption on sports performance, it has also been suggested that caffeine expectancy, the belief of having ingested caffeine, may also evoke improved performance [85]. The placebo effect has been long recognised in medicine [127] but has also received special attention in the sports environment for the purpose of improving athletic performance [128]. The placebo effect associated with caffeine ingestion can be defined as a positive effect on performance after ingesting an inert substance, when participants believe they are ingesting caffeine. Understanding caffeine expectancy is not only important to understanding how caffeine may elicit performance-enhancing effects in real work exercise settings, beneficial effects from expectancy alone would help to circumvent potential side effects of caffeine, promote performance-enhancing effects in non-caffeine responders, any by manipulating an individual’s perception of caffeine performance-enhancing potential, may elicit greater benefits when combined with caffeine pharmacological effects.

For example, a recent paper by Rohloff et al. [129] investigated the placebo effect of caffeine in amateur runners during a 4 km running trial. Participants were tested under three conditions: Control (participants did not ingest any substance); Placebo (a maltodextrin capsule was ingested but participants were informed that they received caffeine); and Caffeine (participants were told they would ingest caffeine, and indeed received 4 mg/kg of caffeine). Performance was improved during the Placebo and Caffeine conditions compared to the Control condition (a 6.89% of improvement in the time employed to cover a 4 km distance), with no differences between Placebo and Caffeine conditions. In another interesting study, Saunders et al. [130] investigated the effects of caffeine supplementation identification on exercise performance in trained cyclists. Those who correctly identified caffeine ingestion improved performance with slightly greater magnitude than the overall effect of caffeine ingestion. Furthermore, participants who incorrectly believed they had ingested tended toward improved performance despite the fact of have been ingested placebo. Interestingly, the correct identification of the placebo trial resulted in harmful effects on performance. However, not all studies support the effect of caffeine expectancy on performance and different deception protocols have been used. The double-dissociation protocol is considered the most suitable, and includes 4 conditions: Placebo (given placebo/told was placebo); Pharmacological (given caffeine/told was placebo); Psychological (given placebo/told was caffeine); and Synergistic (given caffeine/told was caffeine) [131]. Using a double dissociation protocol, Tallis et al. [132] investigated the placebo effect of caffeine and the combined effect of caffeine and expectation on maximal voluntary strength. It was found that caffeine ingestion (5 mg/kg) resulted in improved concentric force in both groups that received caffeine (despite if it was told they ingested caffeine or placebo). Interestingly, performance was poorer when participants believed caffeine ingestion would promote greater benefits.

Results from previous work indicate that the influence of caffeine expectancy would appear to depend on preconceptions and beliefs about the effects of caffeine, and the method of deception employed. In other words, while caffeine per se may result in positive effects on performance, the placebo effect of caffeine supplementation may depend on whether athletes truly believe such supplementation is effective. A study investigated the behaviours and beliefs surrounding caffeine in soccer players [40] and observed widespread use of caffeine by English professional football clubs (97%). Additionally, it was also clear from this study that most athletes believe that caffeine can result in positive effects on athletic performance. For example, 77.7% believe that caffeine can have positive effects on muscular power, and 94.4% believe that it can improve endurance performance to some degree. Therefore, considering that most athletes believe that caffeine can improve sports performance, the placebo effect may have important practical repercussions in elite sports settings, especially considering the importance of marginal gains for elite athletes, where the difference between winning and losing is often minimal and small improvements can have meaningful implications on sporting success.

This issue also raises implications for future scientific studies aiming to assess the effects of caffeine on performance. As highlighted by Halson and Martin [133], a research’s participant preconceptions and beliefs regarding the scientific intervention (e.g., caffeine ingestion) can influence results, as clearly demonstrated by Saunders et al. [130]. However, this is rarely reported in studies, and the effects of expectation on studies with nutritional supplementation are often unclear. As such, future work evaluating the ergogenic effects of caffeine should consider measuring the efficacy of blinding and controlling for this in the analysis. With respect to future studies evaluating the impact of caffeine expectancy, work should focus on directly comparing different methods of deception, evaluating changes in the perception of caffeine over time and considering strategies to manipulate expectancy in individuals that perceive caffeine to have no benefits on exercise performance.

## 9. Adverse Effects of Caffeine on Sleep and Influence on Performance

Whilst acute caffeine consumption has a number of well-established benefits, there is a need to contextualise the typical small but significant improvement in performance from consumption with potential harmful effects. One particular area for focus should be understanding the impact of caffeine on sleep and subsequent performance.

Acute caffeine consumption, particularly when consumed in the late afternoon or evening and when use is infrequent [134], has well-established negative effects on sleep. A recent meta-analysis indicates detrimental effects of low and moderate-dose caffeine (>3 mg.kg^−1^) on several sleep parameters, with caffeine having large negative effects on sleep latency, sleep quality, and post-sleep perception of wakefulness [39]. Sleep loss has been shown to have negative effects on mood [135], reduce several aspects of cognitive and exercise function [136], and impair exercise recovery [137]. Thus, a small acute performance benefit elicited by caffeine consumption could have longer-term negative consequences for performance. Whilst there is some theoretical basis, direct evidence considering the interaction between acute caffeine ingestion, sports performance, sleep and recovery is seldom.

In a population of male distance runners, Ramos-Campo et al. [138] demonstrated that 6 mg.kg^−1^ caffeine had no effect on evening 800 m performance or 800 m performance measured 24-h following the initial trial, despite reporting negative effects on sleep. Although a relationship between caffeine-induced sleep deprivation and performance and recovery was not prevalent here, future work is needed considering the evaluation of different modes and intensities of exercise, the influences of other sleep moderators and the accumulation of effects that may occur from repeat daily caffeine dosing.

## 10. Synergetic Effects of Caffeine & Other Ergogenic Aids

Whilst the ingestion of caffeine tends to be ergogenic, supplementation practices in sport often involve the simultaneous use of several different products [139]. A number of studies suggest that combined caffeine and carbohydrate ingestion offers additive or synergistic performance benefits, or attenuate the effects of fatigue [140,141,142]. One potential mechanism suggested for these benefits is faster intestinal absorption and subsequent increased exogenous carbohydrate oxidation rates during exercise following the ingestion of caffeine and carbohydrate compared with carbohydrate alone [143]. However, Hulston and Jeukendrup [141] reported similar exogenous carbohydrate oxidation rates during exercise when carbohydrate was consumed with or without caffeine.

Caffeine and bicarbonate have also been investigated as supplements that could be used in combination [144,145,146,147,148,149,150], although the effect of this interaction remains unclear. Bicarbonate loading (extracellular buffer) and caffeine (reduced perception of effort) may each enhance the performance of sustained high-intensity exercise. However, Grgic [151] reported that only one [146] out of eight studies on this topic showed additive effects of combining sodium bicarbonate and caffeine. Furthermore, the co-ingestion of these two supplements may increase the risk of gastrointestinal side-effects associated with bicarbonate supplementation and may impair performance [144]. The benefits of the co-ingestion of caffeine and creatine are also unclear. Some studies suggest improvements in exercise performance after caffeine and creatine co-ingestion [152,153]. In contrast, Vandenberghe et al. [154] and Vanakoski et al. [155] reported that the erogenic effects of creatine were unaffected by caffeine consumption. Furthermore, in a recent systematic review, Elosegui et al. [156] concluded that the combination of acute doses of creatine with acute doses of caffeine does not affect exercise performance whilst creatine loading combined with chronic doses of caffeine could interfere with the ergogenic effect of both substances. Trexler et al. [157] reported a greater incidence of gastrointestinal discomfort with the combined ingestion that may decrease absorption of creatine and possibly hinder the creatine loading phase [158]. Another supplement combination is the pairing of nitrate/beetroot juice with caffeine [159,160]. However, there has been no reported benefit from nitrate supplementation, often with a reduction in the benefit of the combined supplementation protocol. In addition, Berjisian et al. [161] reported that the acute co-ingestion of beetroot juice and caffeine did not significantly affect team sport-specific performance.

Overall, the scarcity of studies regarding the co-ingestion of caffeine with additional ergogenic aids makes it difficult to derive a general conclusion [162]. In general, it appears that adding caffeine to carbohydrate is beneficial for performance, although the magnitude and nature of the benefit is yet to be fully elucidated. Further work is required to truly determine whether co-supplementation of caffeine and sodium bicarbonate produces larger ergogenic effects compared with supplementation with either of the supplements alone [151]. Similarly, Buck et al. [163] and Kopec et al. [164] suggest that sodium phosphate and caffeine may improve repeated sprint ability in male team-sports athletes, and therefore warrant additional research to establish the efficacy across over modes of exercise.

## 11. Summary

**Figure 4:**
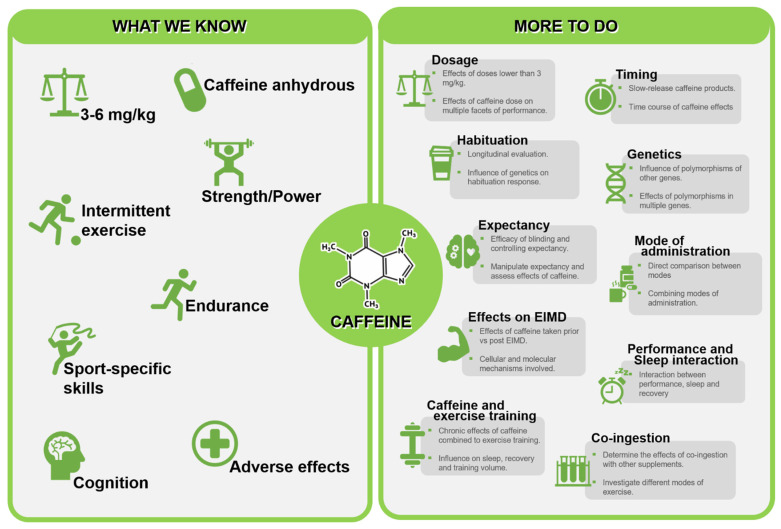
Summary of established effects of caffeine and directions for future investigations. [EIMD = exercise induced muscle damage].

The performance-enhancing potential of acute caffeine consumption is firmly established in the wealth of available literature. However, despite several meta-analyses confirming benefits across several facets important for successful sports performance, key knowledge gaps remain that prevent a precise understanding as to how caffeine can most effectively be used in sport. Given the vast available evidence, identifying meaningful areas of future direction is challenging, and as such, the purpose of the current review was to summarise important key debates within the field and guide the design of future studies to aid in addressing research priorities in this area. Though not an exhaustive list, we identify ten distinct areas of focus as summarised in Figure 4. We recommend future work focus on challenging concepts that have previously been considered to be well established (influence of dose, timing and habituation), areas that have important implications for understating caffeine effectiveness (impact of genotype and caffeine expectancy) and considering key areas that influence application to practice where the evidence base is less established (impact of different modes of administration, efficacy as a supplement for training, interaction between performance and sleep, and synergistic effects with other supplements). Future work in these areas will aid in progressing the current understanding of the effects of caffeine as a performance enhancer.

## Data Availability

Not applicable.
